# Sodium‑glucose co-transporter‑2 inhibitors in the treatment of diabetes with heart failure

**DOI:** 10.1186/s12933-022-01526-4

**Published:** 2022-05-27

**Authors:** Bo Liang, Ning Gu

**Affiliations:** 1 Department of Cardiology, The Second Affiliated Hospital, School of Medicine, Zhejiang University, Hangzhou, China; 2 Nanjing Hospital of Chinese Medicine Affiliated to Nanjing University of Chinese Medicine, Nanjing, China

**Keywords:** Sodium‑glucose co-transporter‑2 inhibitors, Diabetes, Heart failure

## Abstract

2022 AHA/ACC/HFSA guideline for the management of heart failure, which is valuable for clinical decision-making, was recently released. This guideline recommended patients with heart failure with type 2 diabetes sodium‑glucose co-transporter‑2 inhibitors for the management of hyperglycemia and to reduce heart failure-related morbidity and mortality (Class 1, Level A). It is important to note that the source of evidence based on this recommendation is from EMPEROR-Reduced, DAPA-HF, and DECLARE-TIMI 58 and does not include newly published PRESERVED-HF, CHIEF-HF, and EMPEROR-Preserved. Here we reviewed these important trials in order to provide more clinical references for patients with diabetes and heart failure, especially heart failure with preserved ejection fraction.

Recently, the 2022 AHA/ACC/HFSA guideline for the management of heart failure was released [1]. The 2022 heart failure guideline provides recommendations based on contemporary evidence for the treatment of these patients. The recommendations present an evidence-based approach to managing patients with heart failure, with the intent to improve the quality of care and align with patients’ interests. The publication of some landmark clinical trials may rewrite the guideline, but the guideline is not referenced. The 2022 guideline stated in patients with heart failure and type 2 diabetes, the use of sodium‑glucose co-transporter‑2 inhibitors is recommended for the management of hyperglycemia and to reduce heart failure-related morbidity and mortality (Class 1, Level A) [1] based on EMPEROR-Reduced [2], DAPA-HF [3], and DECLARE-TIMI 58 [4]. It is worth noting that empagliflozin/dapagliflozin reduced cardiovascular death and hospitalization for heart failure in patients with heart failure and diabetes, but the ejection fraction was ≤ 40% in EMPEROR-Reduced [2] and DAPA-HF [3], < 45% in DECLARE-TIMI 58 [4], respectively. In other words, empagliflozin/dapagliflozin only reduces cardiovascular outcomes in diabetes with heart failure with reduced ejection fraction and partial mildly reduced ejection fraction. Notably, PRESERVED-HF and CHIEF-HF indicated that dapagliflozin/canagliflozin improved surrogate endpoints (such as Kansas City Cardiomyopathy Questionnaire total symptom score) in patients with diabetes and heart failure (ejection fraction was ≥ 45% in PRESERVED-HF and was not limited in CHIEF-HF) [5, 6]. Moreover, EMPEROR-Preserved was released that empagliflozin reduced the combined risk of cardiovascular death or hospitalization for heart failure in patients with heart failure with preserved ejection fraction (more than 40%), regardless of the presence or absence of diabetes [7]. Ongoing DELIVER will provide new evidence for dapagliflozin in patients with heart failure with preserved ejection fraction (more than 40%) with or without diabetes [8].

Collectively, our current evidence mainly focuses on empagliflozin/dapagliflozin in the treatment of diabetes with heart failure with reduced ejection fraction [9] and empagliflozin in the treatment of diabetes with heart failure with preserved ejection fraction. The role of other types of sodium‑glucose co-transporter‑2 inhibitors in diabetes with heart failure, especially in diabetes with heart failure with preserved ejection fraction, still needs high-quality evidence.

## Data Availability

Data sharing is not applicable to this article as no datasets were generated or analyzed during the current study.
